# Psychological Burden in Uncontrolled Hypertension: Findings from the ERIDANO Multicenter Study

**DOI:** 10.3390/jcm15093309

**Published:** 2026-04-27

**Authors:** Francesca Novello, Fabrizio Vallelonga, Samuel Agostino, Marialaura Di Tella, Agata Benfante, Carlo Aggiusti, Ilaria Fucile, Barbara Maria Colombo, Alessandro Maloberti, Aldo Pende, Massimo Salvetti, Cristina Giannattasio, Costantino Mancusi, Lorys Castelli, Alberto Milan, on behalf of the Eridano Consortium

**Affiliations:** 1 Department of Medical Sciences, University of Turin, 10126 Turin, Italy; francesca.novello@unito.it (F.N.); samuel.agostino@unito.it (S.A.); alberto.milan@ircc.it (A.M.); 2 Division of Internal Medicine, Candiolo Cancer Institute, FPO-IRCCS, 10060 Candiolo, Italy; 3 Department of Psychology, University of Turin, 10124 Turin, Italy; marialauraditella@unito.it (M.D.T.); agatabenfante@unito.it (A.B.); lorys.castelli@unito.it (L.C.); 4 Department of Internal Medicine, ASST Spedali Civili of Brescia, University of Brescia, 25123 Brescia, Italy; carloaggiusti@gmail.com (C.A.); massimo.salvetti@unibs.it (M.S.); 5 Department of Advanced Biomedical Science, Hypertension Research Center, “Federico II” University Hospital of Naples, 80131 Naples, Italy; fucile.ilaria@gmail.com (I.F.); costantino.mancusi@unina.it (C.M.); 6 Emergency Medicine Unit, Department of Internal Medicine, IRCCS AOM Ospedale Policlinico San Martino, University of Genoa, 16132 Genoa, Italy; barbaramaria.colombo@hsanmartino.it (B.M.C.); apende@unige.it (A.P.); 7 Cardiology 4, ASST GOM Niguarda, 20162 Milan, Italy; alessandro.maloberti@ospedaleniguarda.it (A.M.); cristina.giannattasio@unimib.it (C.G.); 8 School of Medicine and Surgery, University of Milan-Bicocca, 20126 Milan, Italy

**Keywords:** uncontrolled hypertension, anxiety, depression, stress, psychological factors

## Abstract

**Background/Objectives:** Uncontrolled hypertension (UH), defined as markedly elevated blood pressure without acute target organ damage, is a clinically relevant condition in which psychological burden remains poorly characterized. We aimed to assess the psychological burden of patients with UH and compare it with that of outpatient hypertensive (HTN) and normotensive (NT) individuals. **Methods:** In this multicenter cross-sectional study, 191 patients with UH, 56 with HTN, and 89 NT individuals were enrolled across six Italian hospitals. Participants completed validated self-report scales assessing anxiety, depressive symptoms, and psychological distress (HADS), perceived stress (PSS-10), and Type D personality traits (DS-14). Statistical analyses included nonparametric group comparisons and multivariable logistic regression with bootstrap resampling. **Results:** UH patients showed significantly higher levels of anxiety, depressive symptoms, and psychological distress than both control groups (all *p* < 0.001). Clinically relevant anxiety was observed in 41.9% of UH patients, compared with 25.0% of HTN and 19.1% of NT participants; depressive symptoms were present in 34.6%, 19.6%, and 12.4%, respectively, and psychological distress in 38.7%, 23.2%, and 14.6%, respectively. Perceived stress was higher in UH than in NT individuals (*p* < 0.001), as were overall Type D personality traits (*p* = 0.016). In multivariable analysis, higher heart rate, smoking, depressive symptoms, dyslipidemia, and prior hypertension were independently associated with UH vs. HTN. **Conclusions:** UH patients exhibit a substantial psychological burden. In this Italian sample, screening for anxiety and depression in patients with UH should be considered in routine clinical practice.

## 1. Introduction

Arterial hypertension is a major global public health challenge and the leading cardiovascular (CV) risk factor, significantly affecting mortality, morbidity, and quality of life [1,2,3].

Within the spectrum of hypertensive presentations, Uncontrolled Hypertension (UH)—corresponding to what has been traditionally defined as hypertensive urgency—refers to marked blood pressure elevation (≥180/110 mmHg) without evidence of acute target organ damage. Patients may experience mild to moderate symptoms and are generally managed with gradual oral blood pressure reduction and outpatient follow-up [1,4].

An increasing number of studies have investigated the psychosocial factors linked to arterial hypertension, with recent evidence indicating a potential association between hypertension and psychological factors [5]. In particular, individuals with anxiety or depressive symptoms appear to exhibit a higher incidence of hypertension compared to asymptomatic individuals [3,6]. Anxiety, depression, and stress have emerged as potential psychological factors involved in the etiopathogenesis of hypertension, although the nature of this relationship remains partially unclear [7]. Furthermore, in recent years, several studies have examined the role of Type D personality (distressed personality) in CV risk.

The most recent evidence suggests that Type D personality is associated with an increased risk of adverse (particularly composite) cardiovascular events, especially in patients with coronary artery disease, while no association with mortality has been demonstrated, and findings remain inconclusive in other clinical populations [8].

In addition, the relationship between psychological factors and blood pressure control has been explored in hypertensive populations, primarily in outpatient settings [9,10].

Although the association between psychological factors and hypertension has been widely investigated, evidence specifically focusing on patients with UH remains scarce, particularly in acute care settings. Moreover, most studies have examined individual psychological dimensions in isolation.

In this context, a multidimensional assessment of psychological factors, including anxiety, depression, distress, perceived stress, and Type D personality, can provide a more comprehensive understanding of the psychological burden associated with UH.

To address this gap, and to our knowledge, this is the first multicenter study comparing patients with UH to both outpatient hypertensive (HTN) and normotensive (NT) individuals using validated psychological scales. This study—part of the ERIDANO (Emergenze Ipertensive e Danno d’Organo) project [11]—aims to characterize the psychological profile and burden of patients with UH.

## 2. Materials and Methods

### 2.1. Study Design

A multicenter, cross-sectional study with a control group was conducted across six Italian hospitals (Turin, Milan, Brescia, Pistoia, Genoa, and Naples), with the Turin center serving as the primary coordinating site. Participant enrollment, initiated in March 2019, is part of the ERIDANO project, aimed at clinically characterizing patients with acute hypertensive disorders and estimating the prevalence of subclinical organ damage [11].

The study included patients aged ≥18 years presenting to the Emergency Department with a symptomatic increase in blood pressure (systolic ≥180 mmHg and/or diastolic ≥110 mmHg) accompanied by at least one symptom suggestive of acute hypertensive organ damage, in accordance with international guidelines [1]. Acute organ damage was systematically assessed in all patients in order to exclude its presence (thus classifying UH) or to confirm Hypertensive Emergencies. Exclusion criteria comprised symptoms not attributable to acute hypertension, elevated blood pressure secondary to trauma, renal colic, or other pain-related conditions (e.g., oncological), as well as lack of informed consent.

For the present analysis, only UH patients were considered with two comparison groups, HTN and NT individuals, that were recruited exclusively at the Hypertension Center in Turin to enable consistent psychological comparisons. The HTN group included outpatients aged ≥18 years with newly diagnosed hypertension who had received their first evaluation at the Turin Center during 2023. The NT group included individuals aged ≥18 years, without a diagnosis of hypertension, with no prior Emergency Department visits for elevated blood pressure, and without disabling chronic diseases (e.g., oncological conditions, neurodegenerative disorders, diabetes with complications, severe cardiovascular or renal disease). NT participants were enrolled through multiple strategies, including announcements disseminated on the Hypertension Center’s social media platforms and flyers distributed in hospital waiting areas.

All participants across the three groups received detailed information about the study, provided written informed consent, and underwent a comprehensive clinical evaluation, including blood pressure measurement and cardiovascular risk stratification.

Self-report psychological questionnaires assessing anxiety, depression, distress, perceived stress, and Type D personality traits were administered to all participants.

### 2.2. Clinical Assessment

Blood pressure was measured following the most recent guidelines of the European Society of Hypertension and the European Society of Cardiology (ESH/ESC) [12,13]. Validated automated sphygmomanometers (e.g., Omron M10-IT, Matsusaka, Kyoto, Japan) were used, and measurements were obtained with patients seated whenever feasible. Three consecutive readings were recorded, and the mean value was considered for statistical analyses.

The following clinical data were collected:

- Cardiovascular risk factors: smoking status, history of arterial hypertension, diabetes mellitus, and dyslipidemia;
- Previous cardiovascular events: coronary artery disease, chronic heart failure, atrial fibrillation (paroxysmal, persistent, or permanent), chronic kidney disease (defined as glomerular filtration rate <60 mL/min/1.73 m^2^), and prior ischemic or hemorrhagic stroke or transient ischemic attack.

### 2.3. Psychological Assessment

All patients, except those presenting with linguistic or cognitive barriers, completed a self-report questionnaire protocol aimed at outlining their psychological profile and exploring potential prognostic implications.

The protocol included the administration of three validated scales. All instruments were administered in their validated Italian versions, which have undergone prior linguistic and cultural adaptation for use in Italian populations. The Hospital Anxiety and Depression Scale (HADS) [14,15], consisting of 14 items rated on a 4-point Likert scale (0–3), was used to assess symptoms of anxiety and depression over the previous week. The scale is divided into two parts: 7 items assess symptoms of anxiety (referred to as the HADS-A subscale) and 7 items assess symptoms of depression (HADS-D subscale). A score of 8 or higher on either part suggests clinically relevant levels of anxiety or depression. In addition, a combined total score of 15 or more across all items indicates the presence of psychological distress. The Perceived Stress Scale (PSS-10) [16,17], a 10-item questionnaire using a 5-point Likert scale (0–4) referring to the past month, measured the level of perceived stress (i.e., the degree to which individuals appraise their life as unpredictable and uncontrollable), where higher scores indicate greater stress perception. Lastly, the Type D Personality Scale (DS-14) [18,19], also composed of 14 items rated on a 5-point Likert scale (0–4), detected the presence of Type D personality, characterized by negative affectivity (NA) and social inhibition (SI), defined by scores ≥10 on both subscales.

### 2.4. Statistical Analysis and Artificial Intelligence Support

Descriptive statistics were reported as the mean and standard deviation (SD) for continuous variables, and frequency and percentage (n, %) for categorical variables. Group comparisons were conducted across three predefined clinical categories: NT, HTN, and UH.

Normality was assessed using the Kolmogorov–Smirnov tests. Since the assumption was violated for all continuous variables, group comparisons were performed using Kruskal–Wallis tests. In addition, visual inspection of distributions and the presence of skewness and unequal group sizes supported the use of nonparametric methods, which are more robust to deviations from normality. Dunn’s post hoc tests with Holm correction were used for multiple testing. For dichotomous outcomes based on clinical cut-offs, differences in prevalence were assessed using Pearson’s chi-square test.

Univariate and multivariable logistic regressions explored associations between group membership and clinical outcomes. A stepwise logistic regression was first conducted to identify the most relevant predictors, followed by a standard Enter method that included the significant predictors. Additionally, a nonparametric bootstrap procedure (1000 samples) was performed to assess the robustness of the estimates. Multicollinearity was checked using Variance Inflation Factor (VIF) and tolerance values, all within acceptable limits. Results are reported as odds ratios (ORs) with 95% confidence intervals.

A priori sample size estimation was based on group means for depression with a common standard deviation, with an estimated effect size of f = 0.29. To achieve 80% power at α = 0.05, a minimum of 123 participants would have been required. The actual sample size (N = 336) ensured good statistical power. Statistical analyses were performed using IBM SPSS Statistics version 30, and graphical representations (box plots) were generated using RStudio (version 2026.01.1 Build 403, Posit Software, PBC) with the ggplot2 package.

During the preparation of this manuscript, the authors employed ChatGPT (GPT-5.3, OpenAI) for the generation of the images included in the graphical abstract.

## 3. Results

### 3.1. Demographic Characteristics

A total of 191 patients with UH were included, alongside a control group consisting of 89 NT volunteers and 56 volunteers with HTN. It should be noted that the DS-14 questionnaire was not completed by all participants; therefore, analyses involving Type D personality were conducted on a reduced sample (N = 182).

The baseline characteristics of the sample, stratified by group, are presented in Table 1. The mean age of the overall sample was 57.6 ± 15.1 years, with a mean Body Mass Index (BMI) of 27.2 ± 5.3 kg/m^2^. BMI, systolic and diastolic blood pressure, and heart rate progressively increased across groups, with the highest values observed in participants with UH. The prevalence of diabetes and dyslipidemia was lowest among NT participants and highest in the UH group.

### 3.2. Anxiety (HADS-A)

Clinically significant anxiety (HADS-A ≥ 8) was more prevalent in the UH group (41.9%) than in HTN (25.0%) and NT participants (19.1%), χ^2^(2) = 16.2, *p* < 0.001. HADS-A scores were 7.0 (IQR = 5.5) in UH, 6.0 (IQR = 4.3) in HTN, and 5.0 (IQR = 5.0) in NT individuals. There were significant differences among groups, H(2) = 22.8, *p* < 0.001, rank η^2^ = 0.06 (95% CI: 0.02–0.13). Post hoc comparisons revealed that UH patients scored significantly higher than both NT (*p* < 0.001) and HTN (*p* = 0.02) individuals (Table 2).

### 3.3. Depression (HADS-D)

The prevalence of clinically significant depressive symptoms (HADS-D ≥ 8) was 34.6% in UH group, 19.6% in HTN, and 12.4% in NT participants, χ^2^(2) = 17.0, *p* < 0.001. Median depression scores were 6.0 (IQR = 5.0) in UH, 4.0 (IQR = 4.0) in HTN, and 4.0 (IQR = 4.0) in NT. Group differences were statistically significant, H(2) = 21.2, *p* < 0.001, rank η^2^ = 0.06 (95% CI: 0.01–0.11). UH participants reported higher scores than both NT (*p* < 0.001) and HTN (*p* = 0.004) groups (Table 2).

### 3.4. Distress (HADS Total)

The prevalence of psychological distress (HADS ≥ 15) was 38.7% in the UH group, 23.2% in HTN, and 14.6% in NT participants, χ^2^(2) = 18.3, *p* < 0.001. Distress scores were 13.0 (IQR = 10.5) in UH, 9.0 (IQR = 8.0) in HTN, and 8.0 (IQR = 7.0) in NT. A significant group difference emerged, H(2) = 27.8, *p* < 0.001, rank η^2^ = 0.08 (95% CI: 0.04–0.15). UH participants scored significantly higher than both NT (*p* < 0.001) and HTN (*p* = 0.004) (Table 2).

### 3.5. Type D Personality

The prevalence of Type D personality was 31.3% in the UH group, 33.9% in HTN, and 21.3% in NT participants (χ^2^(2) = 3.7, *p* = 0.016).

Median DS-14 scores were 20.0 (IQR = 16.0) in UH, 18.5 (IQR = 17.0) in HTN, and 16.0 (IQR = 13.0) in NT participants. A statistically significant difference in scores was found among groups (H(2) = 7.0, *p* = 0.030; rank η^2^ = 0.02, 95% CI: 0.00–0.05).

Post hoc analyses showed higher scores in UH compared to NT participants (*p* < 0.05), while other comparisons were not significant (*p* > 0.05). In a subgroup analysis by sex, no significant difference was observed among males (*p* = 0.341), while a significant difference emerged among females (*p* < 0.05). UH female patients scored significantly higher than NT females (*p* < 0.05). Among women, median DS-14 scores were 20.0 (IQR = 16.0) in UH, 19.0 (IQR = 16.0) in HTN, and 15.0 (IQR = 10.5) in NT (Table 2).

Exploratory analyses modeling Type D personality using continuous negative affectivity, social inhibition, and their interaction did not show significant associations with UH, either in unadjusted or adjusted models. This finding is consistent with recent methodological evidence suggesting that dichotomous Type D classifications may yield spurious associations, often reflecting main effects of underlying emotional components rather than a true combined Type D effect.

### 3.6. Perceived Stress (PSS)

Although no binary classification was applied, perceived stress levels differed significantly among groups, H(2) = 14.1, *p* < 0.001, rank η^2^ = 0.04 (95% CI: 0.005–0.09).

Median PSS scores were 18.0 (IQR = 9.0) in UH, 18.0 (IQR = 10.3) in HTN, and 14.0 (IQR = 8.0) in NT participants.

Post hoc tests showed that patients with UH had significantly higher scores than NT (*p* < 0.001), but not than HTN (*p* = 0.21); no difference was observed between NT and HTN (*p* = 0.09) (Table 2).

**Figure 1:**
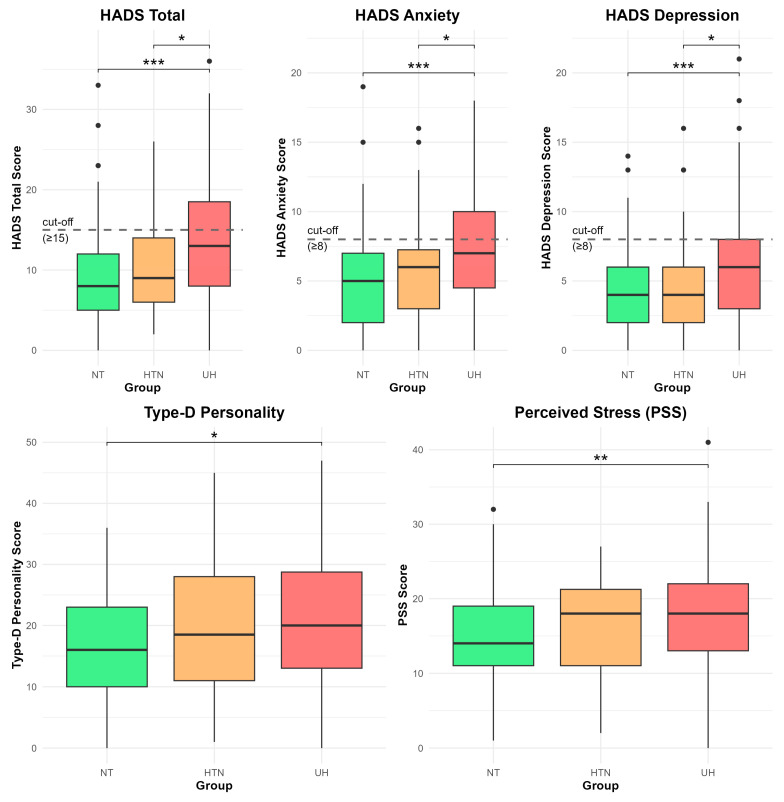
Comparison of Psychological Indicators Across Groups Boxplots show scores for HADS Total, Anxiety, Depression, Perceived Stress (PSS), and Type-D Personality across normotensive, outpatient hypertension, and uncontrolled hypertension. Dashed gray lines represent clinical cut-off thresholds. Kruskal–Holm-adjusted post hoc comparisons followed Wallis tests. Significance: *p* < 0.05 (*), *p* < 0.01 (**), *p* < 0.001 (***). Abbreviations: HADS = Hospital Anxiety and Depression Scale; PSS = Perceived Stress Scale.

Figure 1 presents box plots of psychological scores across groups. Overall, individuals with UH showed significantly higher levels of anxiety, depression, psychological distress, perceived stress, and Type D personality traits compared to participants in the HTN and NT groups.

In addition, women reported significantly higher perceived stress levels than men in all clinical groups (*p* = 0.004). However, the effect of the group (NT vs. UH) is similar in both sexes, with an average difference of approximately 3.5 PSS points between women and men.

### 3.7. Factors Associated with UH

Table 3 summarizes the results of the logistic regression analyses. In the full multivariable model (Enter method), several factors were independently associated with UH, including higher heart rate (OR = 1.08, *p* < 0.001), smoking (OR = 4.60, *p* = 0.013), depressive symptoms (HADS-D; OR = 1.14, *p* = 0.018), and a history of hypertension (OR = 9.41, *p* < 0.001), while dyslipidemia was also positively associated with UH (OR = 2.57, *p* = 0.022). The model demonstrated good calibration (Hosmer–Lemeshow *p* = 0.746), strong explanatory power (Nagelkerke R^2^ = 0.456), and an overall classification accuracy of 80.6%. From a clinical perspective, these associations can be expressed in relative terms. Each 1 bpm increase in heart rate corresponded to an approximately 7–8% higher likelihood of UH. Similarly, each one-point increase in HADS-D score was associated with about a 14% increase in likelihood. Smoking was associated with over a fourfold higher probability, while a history of hypertension was associated with nearly a ninefold increase, indicating a markedly higher likelihood of acute presentation in this sample. Bootstrap resampling (1000 samples) confirmed the stability of the main factors, particularly heart rate, depression, smoking, and hypertension history, with consistent confidence intervals. A stepwise regression approach yielded a comparable model, identifying the same core factors, with similar effect sizes and slightly improved classification accuracy (82.4%). These findings should be interpreted as associations within this specific population.

## 4. Discussion

To the best of our knowledge, this is the first study to comprehensively examine multiple psychological variables in relation to UH, aiming to delineate the psychological profile and burden of these patients.

### 4.1. Demographic and Clinical Aspects

In the ERIDANO sample, UH participants displayed a generally less favorable demographic and clinical profile compared with the NT and HTN groups, with a progressive worsening of CV parameters, comorbidities, and risk factors across the three categories. These patterns indicate that UH individuals represented the most clinically compromised subgroup, characterized by a substantial cardiovascular burden and a likely greater psychological impact. It should be emphasized, however, that these differences largely reflect the recruitment strategy and the intrinsic definition of the three categories, rather than population-based gradients. In particular, full comparability across groups, especially with regard to age, was not entirely achievable within the study recruitment framework. For this reason, they are not further discussed from an epidemiological perspective, but are considered here only as descriptors of the studied sample.

### 4.2. Psychological Burden

The findings of our multicenter cross-sectional study ERIDANO in this Italian sample indicate that patients with UH experience substantially higher levels of anxiety, depression, and overall psychological distress compared with both NT and HTN groups. Clinically relevant symptoms in all three domains were consistently more common in UH participants, whereas they were less frequent in HTN controls and lowest among NT individuals. These gradients underscore a pronounced psychological burden associated with UH in the studied sample.

#### 4.2.1. Anxiety, Depression and Distress

These findings, especially regarding depression, are consistent with previous research [20] reporting an association between UH and a history of depression. However, unlike those reports, which did not find a significant link with anxiety, we observed a clear association between UH and anxiety, with significantly higher anxiety prevalence in UH patients compared to both HTN and NT controls. Similar results have also been reported in other studies [21].

More recent evidence further supports the close relationship between hypertension and depressive symptoms [22,23], showing that individuals with poor blood pressure control have a significantly higher risk of depression compared to those with controlled blood pressure, suggesting a bidirectional association between these conditions [24]. In addition, further recent evidence indicates that anxiety and depression are associated with increased blood pressure variability, a factor linked to higher cardiovascular risk and target organ damage [22,25]. Furthermore, both conditions are common comorbidities in UH population and may be significantly associated with poor blood pressure control, reinforcing the clinical relevance of screening for these conditions in patients with UH.

Despite the extensive research on the relationship between arterial hypertension and disorders such as depression and anxiety, data specifically linking psychological symptoms to UH remain limited and sometimes conflicting. Some data even suggest paradoxically better blood pressure control in individuals with anxiety or depressive symptoms [24]. One study observed no significant association between hypertensive crises (defined as blood pressure values ≥180/120 mmHg) and anxiety–depressive symptoms, even after stratification by age and gender [26]. This lack of association may be related to the monocentric nature of the study, which limits generalizability, as well as its observational non-case–control design. Nonetheless, it represents, to our knowledge, one of the few studies in the literature to specifically address the relationship between hypertensive crises and psychological factors.

Consistent with this, the association between depression and UH has been previously reported [20], although evidence regarding anxiety remains inconsistent [26,27]. Several studies failed to identify a significant relationship between anxiety and hypertensive crises or uncontrolled blood pressure [27].

Our findings add to this literature by providing clear evidence that anxiety is significantly elevated in patients with UH and, importantly, is more prevalent than in both HTN and NT controls. Taken together, these results suggest that anxiety may represent a relevant psychological correlate of UH, rather than a non-specific feature of hypertension.

Depression and anxiety may both contribute to the onset and destabilization of hypertension, including UH, through multiple shared pathophysiological pathways. Both conditions are associated with chronic activation of the sympathetic nervous system and the hypothalamic–pituitary–adrenal (HPA) axis, leading to increased catecholamine release, elevated blood pressure, and impaired vascular homeostasis [21,22,23,27]. In depression, additional factors include endothelial dysfunction and a chronic inflammatory response characterized by elevated cytokines and circulating leukocytes, as well as possible impairment of coronary reserve [22,23,28]. Anxiety is also associated with reduced heart rate variability, a higher risk of ventricular arrhythmias, and a greater likelihood of atherosclerosis development, particularly in phobic anxiety disorders [29]. Heightened renin–angiotensin activity and neuroplastic changes induced by prolonged stress reinforce these alterations [28,30]. Serotonin and norepinephrine dysregulation directly affect cardiovascular function by increasing heart rate, myocardial contractility, and vasoconstriction, promoting blood pressure variability [21].

Finally, maladaptive behavioural patterns often seen in both disorders—such as poor adherence to treatment, physical inactivity, smoking, alcohol consumption, and poor diet—may further amplify CV risk and contribute to blood pressure instability [22,23,28].

Importantly, UH is a multifactorial clinical condition, and acute blood pressure elevations may result from a complex interplay of factors, including treatment non-adherence, acute emotional or physical stress, and other clinical or environmental triggers. Therefore, the associations observed in this study should not be interpreted as causal.

#### 4.2.2. Perceived Stress

Few studies have examined perceived stress in relation to UH or uncontrolled blood pressure, although its role in hypertension has been more extensively investigated. Evidence indicates that each one-point increase in perceived stress score corresponds to approximately a 15% higher risk of developing hypertension [31]. Specific stressors—such as workplace conflicts, job insecurity, personal issues, sexual life, and daily routines—appear to independently influence this risk, underscoring the importance of differentiating stress sources in clinical assessments [31].

In our sample, median perceived stress scores were similar between UH and HTN patients, both significantly higher than NT. Moreover, post hoc tests revealed that perceived stress was significantly higher in UH compared to NT, but not significantly different between UH and HTN, nor between NT and HTN. A plausible hypothesis is that perceived stress exerts a non-uniform effect: without an analysis of stressor sources, total perceived stress may be insufficiently specific to discriminate between UH and HTN.

Recent evidence further supports the role of stress as a relevant risk factor for hypertension, showing that individuals with moderate to high perceived stress levels have a significantly increased likelihood of developing hypertension, even in non-clinical populations [32].

Evidence consistently links stress to elevated blood pressure. Chronic psychological stress nearly doubles hypertension risk, and hypertensive individuals report higher psychosocial stress than normotensive controls [33].

Greater exposure to stressful life events also predicts poorer blood pressure control [34].

Nonetheless, some data suggest a more complex interplay, where perceived stress influences hypertension indirectly through anxiety or depression, potentially assuming a mediating or protective role [7].

Our study aligns with this literature, confirming in this sample the existence of a link between perceived stress and HTN, while emphasizing the need for further research focused on UH.

Evidence indicates that gender may influence the link between perceived stress and hypertension, with men showing a stronger association between high perceived stress and hypertension risk, while no significant relationship emerges in women [35]. In our sample, women reported significantly higher perceived stress than men. Despite this difference, the group effect (NT vs. UH) was similar across genders, with an average gap of approximately 3.5 PSS points between women and men. This pattern suggests that sex differences may primarily affect baseline perceived stress levels rather than clearly modifying its association with blood pressure status. In this context, higher perceived stress in women may reflect greater vulnerability to psychosocial stressors or differences in stress perception and reporting, rather than a differential impact on UH itself.

The older age of our sample may have strengthened the association between perceived stress and female gender, particularly among individuals with UH.

Stress contributes to hypertension through intertwined biological and behavioral mechanisms. Physiologically, activation of the HPA axis, sympathetic stimulation, reduced vagal tone, and immune dysregulation increase heart rate, lower heart rate variability, impair endothelial function, and promote vasoconstriction, thereby elevating hypertension risk [7,36,37,38]. Excess secretion of stress hormones such as glucocorticoids and catecholamines further contributes to cardiovascular and immune dysfunction.

On the behavioral level, stress can trigger maladaptive responses, promoting unhealthy habits which, over time, contribute to increased hypertensive risk [31,38].

#### 4.2.3. Type D Personality

Type D personality is included among CV risk factors to be assessed in clinical practice in the European Guidelines on Cardiovascular Disease Prevention [39]. Several studies [8,40,41] have examined the relationship between this personality trait and CV risk.

Early studies involving large general population and clinical samples (patients with cardiac disease and hypertension) confirmed this pattern, showing particularly high rates in hypertensive and coronary artery disease groups [18]. These findings are consistent with those observed in our study, which showed a higher prevalence of Type D personality in subjects with HTN and UH compared to NT individuals. However, the prevalence rates observed in our hypertensive cohort were lower than those reported in the previous study.

Evidence has shown that Type D personality is twice as prevalent among Italian hypertensive individuals compared to normotensive individuals, and that Type D individuals have a higher incidence of hypertension [42].

Recent evidence from case–control studies has shown a higher prevalence of Type D personality among hypertensive individuals compared with the general population [43]. In parallel, individuals with Type D personality appear to have a higher likelihood of hypertension. These findings are in line with our results, which also show a higher prevalence of Type D personality in HTN and UH compared with NT individuals.

However, when Type D personality was modeled using its continuous components (negative affectivity, social inhibition, and their interaction), no significant associations were observed, in line with recent evidence suggesting that dichotomous Type D classifications may reflect underlying emotional traits rather than a true combined Type D effect [44].

These findings are consistent with our results, which show a higher prevalence of Type D personality in HTN and UH compared with NT individuals. The lower prevalence observed in our cohort may reflect differences in sample characteristics, suggesting variability across populations.

Furthermore, Type D personality appears to be associated with reduced blood pressure reactivity to acute stress, particularly in women [45]. In our sex-disaggregated analysis, no significant differences emerged among men, whereas women with UH exhibited significantly higher Type D scores than NT females. This finding highlights a sex-specific pattern, suggesting that the association between Type D personality and UH may be more pronounced in women. Notably, reduced physiological stress reactivity does not necessarily imply lower psychological vulnerability. This observation is consistent with evidence indicating that women tend to report higher levels of internalizing psychological symptoms, such as anxiety and depression, compared to men, as well as a greater vulnerability to psychosocial stressors [46]. These dimensions are closely related to the negative affectivity component of Type D personality. In addition, studies in cardiovascular populations have shown that Type D personality is associated with higher levels of anxiety and depression and that women may exhibit higher levels of social inhibition [47]. In this context, Type D personality may reflect a broader and more persistent vulnerability to psychological distress, which appears to be more pronounced in female patients. While previous studies have mainly focused on stress reactivity, our findings emphasize the relevance of stable personality traits, suggesting that Type D personality may represent a psychological vulnerability associated with UH, particularly in women.

Although the potential association between Type D personality and hypertension is of considerable interest, the existing literature presents conflicting data.

Some studies [48,49] have reported low prevalence of Type D personality among hypertensive subjects. This discrepancy may be attributed to the fact that the sample predominantly consisted of elderly individuals, suggesting a possible decline over time in specific personality traits. Our cohort’s median age—63 years for HTN and 59 years for UH—may partly explain these discrepancies.

Importantly, no prior studies have specifically linked perceived stress and Type D personality with UH or, as it is often defined, with conditions of UH.

Type D personality is strongly linked to chronic stress and can disrupt regulation of stress-related hormones such as cortisol and norepinephrine via the HPA axis, autonomic nervous system, and sympatho-adrenal-medullary system [43,50]. It has also been associated with impaired autonomic regulation, including reduced heart rate variability, delayed cardiovascular recovery, and processes related to atherosclerosis, thereby increasing cardiovascular risk through effects on blood pressure, lipid metabolism, and inflammation [8,50].

Individuals with Type D personality also tend to engage in unhealthy behaviors, further amplifying their cardiovascular risk [50].

A plausible mechanism linking Type D personality to UH is medication non-adherence. Type D personality has been associated with poorer adherence across different chronic conditions, including chronic myeloid leukemia, peritoneal dialysis, coronary artery disease, and heart failure [51,52,53,54]. In hypertensive patients, non-adherence to antihypertensive therapy is a major determinant of inadequate blood pressure control, and non-adherent individuals are significantly more likely to have uncontrolled blood pressure [55,56].

Taken together, these findings suggest that reduced adherence may represent a behavioral pathway linking Type D personality to poor blood pressure control. In the context of UH, which is often related to suboptimal long-term blood pressure management, non-adherence may contribute to acute elevations in blood pressure in the absence of organ damage. However, adherence was not directly assessed in our study, and this hypothesis should be specifically investigated in future studies on UH.

### 4.3. Clinical Implications

These results suggest that psychological evaluation and appropriate interventions should be integrated into routine management of hypertensive patients, particularly those with UH, consistent with the bio-psycho-social model, which emphasizes the importance of a multidimensional approach to patient care [57].

In particular, the use of brief and validated screening tools (e.g., HADS or PSS) in Emergency Department settings or during early outpatient follow-up may represent a feasible strategy to systematically identify patients with clinically relevant psychological distress, allowing timely referral to psychological support and preventive cardiovascular care.

A recent Cochrane systematic review [58] of 21 studies highlighted that various psychological interventions—including cognitive behavioral therapy, relaxation techniques, psychoeducation, and mindfulness-based stress reduction—yield significant beneficial effects on anxiety and depressive symptoms in patients with ischemic heart disease and heart failure.

Consistently, interventions such as mindfulness or stress management training in hypertensive women have shown reductions in anxiety, depression, and blood pressure, with effects sustained for several weeks after treatment [59].

Based on these findings, patients with UH and significant psychological burden may benefit from structured referral pathways to psychological or psychiatric services, ideally within an integrated multidisciplinary care model involving cardiovascular and mental health professionals. Such an approach may also improve treatment adherence and contribute to better long-term blood pressure control.

### 4.4. Strengths and Limitations

This study presents several strengths. First, the multicenter design enhances the generalizability of our findings within the Italian context and increases the representativeness of the national sample. Moreover, the inclusion of three groups (NT, HTN, and UH) enabled the assessment of psychological specificities associated not only with the presence of hypertension but, more importantly, with the acute form represented by UH. Additional relevant aspects include the use of validated instruments and the integrated measurement of medical and psychological variables, offering a multidimensional view of the patients’ profile. Moreover, the use of stratified analyses by sex and robust statistical techniques (stepwise and enter logistic models, bootstrap resampling) strengthened the internal validity and reliability of the associations identified.

Some minor limitations should be acknowledged.

First, its cross-sectional design precludes any causal inference and does not allow assessment of temporal or directional relationships between psychological factors and blood pressure levels, although a longitudinal follow-up is currently planned. In addition, we did not assess potentially relevant factors such as treatment adherence, acute stressors, or other transient triggers that may contribute to blood pressure decompensation. Given the multifactorial nature of uncontrolled hypertension, these unmeasured variables may have influenced the observed associations.

Additionally, potential confounders, such as psychotropic medication use (e.g., antidepressants, anxiolytics) or differences in antihypertensive regimens, were not controlled for and could influence both psychological status and blood pressure control.

Furthermore, all psychological measures were based on self-report questionnaires, which are subject to reporting bias and social desirability effects. Participants may under- or over-report symptoms, potentially inflating or attenuating the observed associations.

Another limitation concerns the recruitment strategy of the control groups. Normotensive (NT) participants were enrolled through voluntary response methods (e.g., flyers and social media announcements), which may introduce selection bias by preferentially including individuals who are more health-conscious or psychologically engaged. This may limit the representativeness of the NT group and potentially affect comparisons with clinical populations.

Moreover, the geographic composition of the samples: UH patients were recruited from multiple centers across Italy, whereas the HTN and NT control groups were enrolled solely at the Turin center. This discrepancy may affect the comparability of the groups and the generalizability of findings to other regions. Full demographic matching—particularly for age—was methodologically challenging, as recruiting normotensive individuals over 60 years old is inherently difficult. While heterogeneous recruitment strategies may introduce selection biases, these approaches were necessary to achieve sufficient sample sizes, and the inclusion of covariates in regression models, along with sex-stratified analyses, helps mitigate potential imbalances and strengthens the robustness of the observed associations. Nevertheless, between-group comparisons should still be interpreted with caution.

## 5. Conclusions

This multicenter cross-sectional study offers novel insights into the psychological characteristics of patients with uncontrolled hypertension (UH), revealing significantly higher levels of anxiety, depressive symptoms, and psychological distress compared to both hypertensive and normotensive individuals. Although no significant differences emerged between UH and HTN in terms of perceived stress or Type D personality, UH patients showed a distinct psychological burden, particularly in comparison to normotensive controls.

These findings reinforce the importance of incorporating psychological screening and support into the management of hypertensive patients, particularly those presenting with UH. While causal relationships cannot be inferred due to the cross-sectional design, early identification and targeted intervention on psychological distress may offer valuable opportunities for comprehensive CV prevention.

Future longitudinal studies are warranted to clarify causal pathways and assess whether psychological interventions can improve both mental well-being and blood pressure control in this population.

## Figures and Tables

**Table 1:** Baseline Characteristics of the Sample by Group.

Variable	All Sample (N = 336)	Normotensive (N = 89)	Outpatient Hypertension (N = 56)	Uncontrolled Hypertension (N = 191)	*p*-Value
Age (years)	57.6 ± 15.1	49.5 ± 16.4	63.8 ± 9.1	59.6 ± 14.4	0.001 *,†
Sex (female)	179 (53.3%)	49 (55.1%)	37 (66.1%)	93 (48.7%)	0.067
BMI (kg/m^2^)	27.2 ± 5.3 ^b^	24.3 ± 3.3	26.8 ± 4.5	28.7 ± 5.7	0.001 *,†,‡
Systolic BP (mmHg)	138.8 ± 21.7	122.5 ± 14.0	138.0 ± 18.8	147.1 ± 21.2	0.001 *,†,‡
Diastolic BP (mmHg)	82.4 ± 13.3	74.7 ± 8.9	80.8 ± 11.4	86.7 ± 13.8	0.001 *,†,‡
Heart rate (bpm)	72 ± 13	69 ± 12	66 ± 8	75 ± 13	0.001 *,‡
Diabetes mellitus	33 (9.9%) ^a^	2 (2.3%)	4 (7.1%)	27 (14.2%)	0.006
Dyslipidemia	248 (73.8%) ^b^	83 (33.5%)	31 (12.5%)	134 (54.0%)	0.001
History of stroke	9 (2.7%) ^c^	0 (0.0%)	1 (1.8%)	8 (4.2%)	0.115
Heart failure	4 (1.2%) ^c^	0 (0.0%)	0 (0.0%)	4 (2.1%)	0.213
Ischemic heart disease	22 (6.6%) ^c^	0 (0.0%)	0 (0.0%)	22 (11.6%)	0.001
Smoking (current/former)	80 (23.9%) ^c^	15 (16.9%)	4 (7.1%)	61 (32.1%)	0.001

Data are presented as n (%) for binary variables (presence = 1), mean ± standard deviation. Abbreviations: BMI = Body Mass Index; BP = Blood Pressure. ^a^ Missing data for 2 participants. ^b^ Missing data for 7 participants. ^c^ Missing data for 1 participant. * = *p* < 0.05 between NT and UH. † = *p* < 0.05 between NT and HTN. ‡ = *p* < 0.05 between HTN and UH.

**Table 2:** Frequency of Clinical Cut-off Cases Across Groups (Chi-Square Tests).

Variable (Cut-Off ≥)	Normotensive n/N (%)	Outpatient Hypertension n/N (%)	Uncontrolled Hypertension n/N (%)	χ^2^ (df)	*p*-Value
Anxiety (HADS-A ≥ 8)	17/89 (19.1%)	14/56 (25.0%)	80/191 (41.9%)	16.21 (2)	<0.001
Depression (HADS-D ≥ 8)	11/89 (12.4%)	11/56 (19.6%)	66/191 (34.6%)	16.96 (2)	<0.001
Distress (HADS Total ≥ 15)	13/89 (14.6%)	13/56 (23.2%)	74/191 (38.7%)	18.30 (2)	<0.001
Type D Personality (NA ≥ 10 and SI ≥ 10)	19/89 (21.3%)	19/56 (33.9%)	57/182 (31.3%) *	3.66 (2)	0.016

Values represent number of participants above cut-off over total in group (n/N), with corresponding percentages. Chi-square tests are based on Pearson’s χ^2^ statistic, 2-sided, df = 2. * Lower total number of patients due to incomplete administration of the DS-14 scale. Abbreviations: HADS = Hospital Anxiety and Depression Scale; HADS-A = Hospital Anxiety and Depression Scale-Anxiety; HADS-D = Hospital Anxiety and Depression Scale-Depression; NA = Negative Affectivity; SI = Social Inhibition.

**Table 3:** Multivariable logistic regression results and model performance.

**Factors**	**OR Stepwise (95% CI)**	**OR Enter (95% CI)**	**OR Bootstrap (95% CI)**
BMI (kg/m^2^)	1.09 (1.01–1.17)	1.09 (1.01–1.17)	1.09 (1.01–1.20)
Depression (HADS-D)	1.14 (1.02–1.27)	1.14 (1.02–1.28)	1.14 (1.02–1.35)
Clinical heart rate (bpm)	1.07 (1.04–1.11)	1.08 (1.03–1.12)	1.08 (1.04–1.13)
Smoking	4.95 (1.52–16.14)	4.60 (1.38–15.32)	4.60 (1.96–31.85)
Dyslipidemia	2.56 (1.14–5.70)	2.57 (1.15–5.71)	2.56 (1.07–6.96)
Hypertension	9.53 (3.75–24.24)	9.41 (3.63–24.41)	9.41 (3.71–36.18)
**Model**	**Nagelkerke R^2^**	**Accuracy (%)**	
Stepwise	0.448	82.4	
Enter	0.456	80.6	
Bootstrap	0.456 *	80.6	

Outcome: Uncontrolled hypertension (1) vs. Outpatient hypertension (0). OR = Odds Ratio; CI = Confidence Interval. * Bootstrap based on 1000 resamples.

## Data Availability

The data presented in this study are available on reasonable request from the corresponding author.

## Abbreviations

The following abbreviations are used in this manuscript:

UH	Uncontrolled Hypertension
HTN	Outpatient Hypertensive
NT	Normotensive
CV	Cardiovascular
ERIDANO	Emergenze Ipertensive e Danno d’Organo
HADS	Hospital Anxiety and Depression Scale
HADS-A	Hospital Anxiety and Depression Scale-Anxiety
HADS-D	Hospital Anxiety and Depression Scale-Depression
PSS-10	Perceived Stress Scale-10
DS-14	Type D Personality Scale-14
NA	Negative Affectivity
SI	Social Inhibition
SD	Standard Deviation
VIF	Variance Inflation Factor
OR	Odds Ratio
HPA	Hypothalamic–Pituitary–Adrenal

## Appendix A

Authors of the Eridano Consortium: Dario Leone, Salvatore Fragapani, Giulia Bruno, Giulia Mingrone, Martina Sanapo, Anna Colomba, Jacopo Ligato, Elvira Fanelli, Lorenzo Airale, Arianna Paladino, Enrico Lupia, Fulvio Morello, Maria Tizzani, Carmine De Luca, Raffaele Izzo, Antonella Ioverno, Paola Sormani, Michela Algeri, Nicola Nesti, Franco Cipollini and Nicola De Luca.
